# An In Vitro Comparison of Costimulatory Domains in Chimeric Antigen Receptor T Cell for Breast Cancer Treatment

**DOI:** 10.1155/2022/2449373

**Published:** 2022-11-22

**Authors:** Nattarika Khuisangeam, Sirirut Jewmoung, Rattapoom Thaiwong, Suparat Tudsamran, Nattiya Hirankarn, Koramit Suppipat, Supannikar Tawinwung

**Affiliations:** ^1^Department of Microbiology, Faculty of Medicine, Chulalongkorn University, Bangkok 10330, Thailand; ^2^Chulalongkorn Comprehensive Cancer Center, King Chulalongkorn Memorial Hospital, Bangkok 10330, Thailand; ^3^Cellular Immunotherapy Research Unit, Chulalongkorn University, Bangkok 10330, Thailand; ^4^Department of Pharmacology and Physiology, Faculty of Pharmaceutical Sciences, Chulalongkorn University, Bangkok 10330, Thailand

## Abstract

Adoptive cellular therapy with chimeric antigen receptor (CAR) T cells has emerged as a potential novel treatment for various cancers. In this study, we have generated CAR T cells targeting mucin-1 (MUC1), which is an aberrantly glycosylated antigen overexpressed on breast cancer cells. Two different signaling domains, including CD28 and 41BB, were incorporated and directly compared the superiority of different costimulatory signals. Two different CAR MUC1 constructs were transduced into primary T cells and evaluated their characteristics and antitumor activities against MUC1^+^ cancer cells. CAR MUC1 T cells showed high transduction efficiency and antigen specificity toward MUC1^+^ cancer cell lines and primary breast cancer cells. When coculturing with target cells, the transduced cells exhibited potent antitumor activity in vitro and secrete proinflammatory cytokines. Upon antigen stimulation, incorporation of the 41BB signaling domain was able to improve T cell proliferation and reduce surface PD1 expression and the upregulation of suppressive cytokines, when compared with CAR MUC1 containing the CD28 domain. Our findings show that CAR T cell targeting MUC1 can be effective against MUC1^+^ breast cancer cell and support the further development of CAR MUC1 T cells containing 41BB signaling in preclinical and clinical studies of breast cancer treatment.

## 1. Introduction

Breast cancer has the highest incidence rate and remains to be one of the leading causes of cancer mortality among women [1, 2]. Molecular subtyping of breast cancer based on expression of molecular markers has provided clinical benefits for predicting prognosis and improving treatment effects [3, 4]. Although significant advances in surgery, radiation, chemotherapy, endocrine therapy, and molecular targeted therapy have been associated with favorable clinical outcomes for various molecular subtypes, many cancers have become resistant to therapy, and some subtypes lacking molecular targets, such as triple-negative breast cancer, remain difficult to treat [5]. Tumor-immune interaction has been considered to play a prominent role in cancer progression, and a wide range of immunotherapies, including immune checkpoint inhibitors, cancer vaccines, and adoptive cell therapies, have been developed to advance breast cancer treatment [6, 7]. Recently, adoptive cell transfer with genetically modified chimeric antigen receptor (CAR) T cells has emerged as a novel promising treatment with outstanding clinical outcomes in hematologic malignancies [8], prompting efforts to extend its benefits to solid tumors. CAR T cells targeting different tumor-associated antigens are also under investigation in both preclinical and clinical studies of breast cancer treatment [9].

Mucin-1 (MUC1) is a type I transmembrane protein, uniformly overexpressed on breast cancer, and its expression serves as a predictive marker for metastatic progression and poor prognosis [10]. The extracellular domain of MUC1 (MUC1-N) is known to contain a variable number tandem repeat (VNTR) consisting of 20–21 amino acid sequence repeats. In normal cells, MUC1 is extensively O-glycosylated at serine (Ser) and threonine (Thr) residues in the VNTR [11, 12]. This hyperglycosylation masks the MUC1 peptide core from proteolytic cleavage and stabilizes membrane MUC1. In malignant cells, however, tumor-associated MUC1 (tMUC1) differs from MUC1 that expressed on normal cell in its structure and distribution. tMUC1 expressed up to 10 times higher than in normal tissues and aberrantly glycosylated, which, in turn, impacts its subcellular localization and oncogenic signaling including the regulation of genes related to cell proliferation, invasion, apoptosis, drug resistance, and angiogenesis [11, 13–15]. In breast cancer, MUC1 is highly sialylated because of increased expression of *α*2,3-sialyltransferase [16, 17]. This results in premature termination of glycopeptide bonds and the exposure of the MUC1 core peptide. Therefore, antibodies that are designed to target epitopes within the MUC1 core peptide or the altered glycopeptide epitopes are more likely to bind to tMUC1, and not to MUC1, on normal cells, thus rendering them an attractive candidate molecule for cancer-specific immunotherapies [18]. CAR targeting MUC1 derived from monoclonal antibodies specific to the tumor-associated MUC1 glycoforms was developed, and its antitumor efficacy has been demonstrated in multiple epithelial cancers including ovarian cancer, adenocarcinoma, leukemia, pancreatic cancer, head and neck squamous cell carcinoma, cholangiocarcinoma, and breast cancer [19].

Generally, CAR is encoded by a single gene consisting of an antigen-binding domain, transmembrane domain, and an intracellular domain. The intracellular domain provides the T cell activating function and typically includes signal transduction of CD3*ζ* containing three immunoreceptor tyrosine-based activation motifs fused with signal domains of costimulatory molecules, of which CD28 and 41BB have been used for second-generation CAR T cells in clinical trials and approved commercial products. Comparison of the consequences of incorporating either 41BB- or CD28-derived costimulatory domains has been widely examined. However, the superiority of CARs containing either 41BB or CD28 costimulatory domain has not been consistently observed among different targets, and CAR constructs as reviewed by Cappell and Kochenderfer [20]. These differences, however, may be influenced by other factors including various experimental conditions and the specific CAR constructs designed for the scFv or hinge/transmembrane domain region. Therefore, preclinical studies to directly compare the effects of different costimulatory domains in identical CAR constructs are warranted.

In this present study, we generated second-generation CAR T cells targeting tumor-associated MUC1 for breast cancer and evaluated the effects of two costimulatory molecules on antitumor activities and immunophenotypes related to CAR T cell persistence. As per our findings, we found that CAR MUC1 containing 41BB has ameliorated the upregulation of PD-1 expression and reduced the production of immunosuppressive cytokines compared with the incorporation of a CD28 signal. This preclinical evaluation provides evidence for the selection of a particular MUC1-targeted CAR construct for further in vivo and clinical studies of breast cancer treatment.

## 2. Materials and Methods

### 2.1. Primary Cell and Cell Lines

The MCF-7 breast cancer cell line, CaOV3 ovarian cancer cell line, and 293T human kidney embryonic cell line were obtained from the American Type Culture Collection (Rockville, MD). Cancer cell lines were cultured in Dulbecco's modified Eagle medium (DMEM, GE Healthcare Life Sciences) supplemented with 10% heat-inactivated fetal bovine serum (FBS) (Hyclone, Waltham, MA) and 2 mM L-GlutaMAX (Gibco BRL Life Technologies, Inc.). Tissue biopsies of proven breast cancer patients were obtained from tissue bank. Breast cancer tissue biopsies were dissociated to single-cell suspensions of primary breast cancer cell using a human Tumor Dissociation Kit (Miltenyi Biotec). Tumor dissociation was performed according to the manufacturer's protocol. Peripheral blood mononuclear cells (PBMCs) were obtained from healthy volunteers after informed consent. All procedures were done following the protocol approved by the Institutional Review Board of the Faculty of Medicine, Chulalongkorn University (IRB NO.437/62). All cells were maintained in a sterile humidified atmosphere containing 5% carbon dioxide (CO_2_) at 37°C. All experiments were performed in accordance with the relevant guidelines and regulations.

### 2.2. Generation of CAR Constructs and Retroviral Vectors

The second-generation CAR MUC1 constructs consisted of an anti-MUC1 scFv sequence derived from the published monoclonal antibody clone HMFG2 [21]. The HMFG2 scFv was linked with IgG2-derived CH3 and the CD28 transmembrane domain, followed by the endodomain, which consisted of the intracellular domain of costimulatory 41BB or CD28 fused with the CD3 zeta (*ζ*) chain. The codon-optimized CAR plasmids were then synthesized in an SFG retroviral backbone. To produce the retroviral vector, human kidney embryonic 293T cells were cotransfected with SFG encoding the second-generation CAR MUC1 plasmid, the plasmid containing a MomLV gag-pol sequence, and the RD114 envelope plasmid, using GeneJuice transfection reagent (Novagen, Billerica, MA). Retroviral supernatants were then collected at 48 and 72 hours after transfection.

### 2.3. Generation of CAR MUC1 T Cells

PBMCs were isolated from peripheral blood (PB) collected from healthy donors in sodium heparin tubes using Ficoll-Paque Premium (Cytiva, Global Life Sciences, Marlborough, MA, USA). To selectively expand T cells by using OKT3 and CD28 activation, PBMCs were activated with OKT3 (1 mg/ml) (Miltenyi Biotec, Bergisch Gladbach, Germany) and CD28 (1 mg/ml) (BD Bioscience) precoated plates for 72 h with recombinant human IL-2 (rhIL-2, 50 U/ml) supplementation. Cells were cultured in complete media (RPMI-1640 and 45% Click's medium, Sigma-Aldrich, Inc., St. Louis, MO, USA). To generate CAR T cell, the retroviral supernatant was added to a 24-well nontissue culture-treated plate precoated with recombinant fibronectin fragment (FN CH-296; RetroNectin; Takara Shuzo, Otsu, Japan) and centrifuged at 2000 g for 90 min. The viral supernatant was removed, and OKT3/CD28-activated T cells were resuspended in complete media supplemented with IL-2 (50 U/ml) and added to the wells. Transduction efficiency was measured 4 days posttransduction by flow cytometry. OKT3/CD28-activated T cells were added to the well which were precoated with RetroNectin without viral supernatant and included as NT control cells.

### 2.4. Flow Cytometry

Transduced cells were stained with fluorochrome-conjugated monoclonal antibodies for 15 min at 4°C. The cells were washed twice with 1x PBS supplemented with 1% FBS. For cell surface marker analysis, the following antibodies were used: CD56-PE (HCD56/Cat#130-114-551), CD4-FITC (OKT4/130-114-531), CD8-APC (SK1/130-110-679), CD45RO-APC (UCHL1/130-113-556), CD62L-FITC (DREG-56/130-112-077), CD25-APC (BC96/130-113-284) (Miltenyi Biotec, Bergisch Gladbach, Germany), PD1-FITC (EH12.2H7/329904), LAG-3-FITC (11C3C65/369308), TIM-3-APC (F38-2E2/345012), and CD3-PerCP (clone OKT3/317336) (BioLegend, San Diego, CA, USA). The memory phenotypes were defined as naïve (TN; CD3^+^CD45RO^−^CD62L^+^), effector memory (TEM; CD3^+^CD45RO^+^CD62L^−^), central memory (TCM; CD3^+^CD45RO^+^CD62L^+^), and terminal effector (TE; CD3^+^CD45RO^−^CD62L^−^) T cells. For transduction efficiency of transduced cells, AF647-conjugated anti-human IgG (H+L) (Jackson ImmunoResearch/109-607-003) was used to detect CAR expression. Flow cytometry was performed using a BD Accuri™ C6 Plus Flow Cytometer (BD Bioscience), and data were analyzed by the FlowJo V10.7.1 software (FlowJo).

### 2.5. Coculture Experiments

The antitumor activity of CAR T cells was examined by coculturing CAR T cells with the eGFP-FFLuc expressing MCF-7 cell line at effector/target (E:T) ratios of 1 : 1, 1 : 2, and 1 : 5 in DMEM containing 10% FBS and 2 mM L-GlutaMAX without additional cytokines for 48 and 72 hours. Cells were harvested and quantified by flow cytometry using CountBright™ Absolute Counting Beads (Invitrogen), and the acquisition was ceased at 5,000 beads. 7-AAD (BD Bioscience) was then added to exclude dead cells. The percentage of inhibition was calculated using the following formula:

(1) $$\begin{array}{c} \%\text{Inhibition}=100-\left(\frac{\text{number of Target }\left(\text{experiment}\right)}{\text{number of Target }\left(\text{Target alone}\right)}\times 100\right). \end{array}$$

After coculturing for 72 h, harvested T cells were analyzed for the expression of activating and exhaustion markers using flow cytometry following staining with CD25-APC, PD1-FITC, LAG-3-FITC, and TIM-3-APC antibodies.

### 2.6. Cytokine Detection

CAR T cells were cocultured with MUC1^+^ cancer cells at an E:T ratio of 1 : 1 for 72 h. Culture supernatants were then collected, and dead cells were removed and stored at −20°C. Quantification of cytokines was analyzed by flow cytometry using the BD™ CBA Human Th1/Th2 Cytokine Kit II (BD Bioscience), and the data were analyzed by the FCAP Array version 4 software (BD Bioscience) [22].

### 2.7. Statistical Analysis

The results were reported as mean ± SEM. All statistical analyses were performed using GraphPad Prism version 8 (GraphPad Software). One-way and two-way ANOVA were used to determine statistical significance between and among groups. *P* values less than 0.05 were considered statistically significant.

## 3. Results

### 3.1. Generation of Second-Generation CAR T Cells Directed against Tumor-Associated MUC1

**Figure 1:**
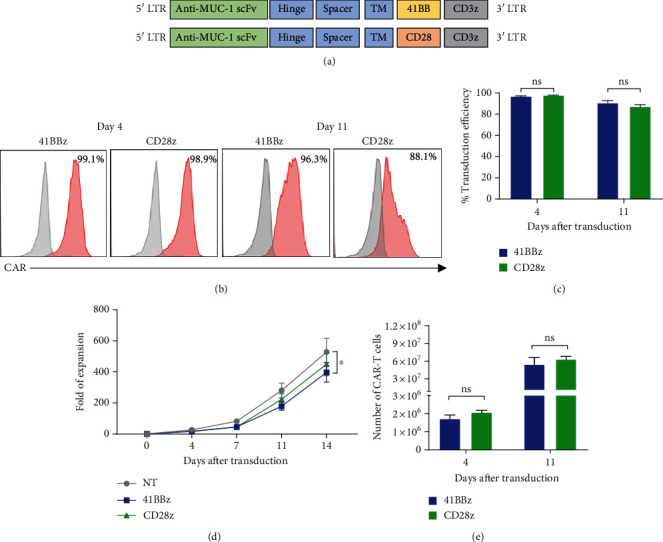
Generation of CAR T cell targeting tumor associated MUC1. (a) Schematic of CAR MUC1 constructs with either CD28*ζ* or 4-1BB*ζ* costimulatory signaling domain. (b) Representative histogram demonstrating CAR.MUC1-41BB*ζ* and CAR.MUC1-CD28*ζ* expression on day 4 and day 11 after transduction. (c) Transduction efficiency of CAR.MUC1-41BB*ζ* and CAR.MUC1-CD28*ζ*. Data are shown as mean ± S.E.M from 6 independent donors. (d) Ex vivo expansion of CAR.MUC1-41BB*ζ* and CAR.MUC1-CD28*ζ*. Cell numbers were determined by trypan blue exclusion assay on days 0, 4, 7, 11, and 14 after T cell transduction; data are presented as fold expansion and shown as mean ± S.E.M (*n* = 16). (e) The number of CAR^+^ cell on days 4 and 11 after transduction, the data are shown as mean ± S.E.M from six donors. Significance was determined by two-way ANOVA, ^∗^ for *P* < 0.05, ^∗∗^ for *P* < 0.01, and ^∗∗∗^ for *P* < 0.001.

To target tumor-associated MUC1 on breast cancer cells, we have generated second-generation CAR MUC1 T cells with two differential costimulatory signals, that is, 41BB and CD28, and determine their immune characteristics and *in vitro* antitumor activities.  Figure 1(a) illustrates the components of the two CAR constructs. Both CAR.MUC1-41BB*ζ* and CAR.MUC1-CD28*ζ* T cells exhibited transduction efficiencies of more than 90% on day 4 after transduction (Figures 1(b) and 1(c)). The CAR T cells were expanded in the presence of IL-2 (50 U/ml) for 1 week, and CAR expression was then reanalyzed. We observed the transduction efficiency of 90.6% ± 2.6% for CAR.MUC1-41BB*ζ* and 87.15% ± 2.36% for CAR.MUC1-CD28*ζ*, in which there was no statistically significant difference between the two constructs (*P* = 0.3671) (Figures 1(b) and 1(c)). Total T cell expansion levels were then determined. On day 14 of cultivation, the fold expansion of CAR T cells was 396.6 ± 62.5 and 449.5 ± 72.9 for CAR.MUC1-41BB*ζ* and CAR.MUC1-CD28*ζ* T cells, respectively (Figure 1(d)). On day 11, at which time the cells were harvested for further experiments, the absolute number of CAR.MUC1-41BB*ζ* and CAR.MUC1-CD28*ζ* T cells was 5.45 ± 1.29 × 10^7^ and 6.32 ± 0.57 × 10^7^, respectively, which is not statistically different (*P* < 0.0001) (Figure 1(e)).

**Figure 2:**
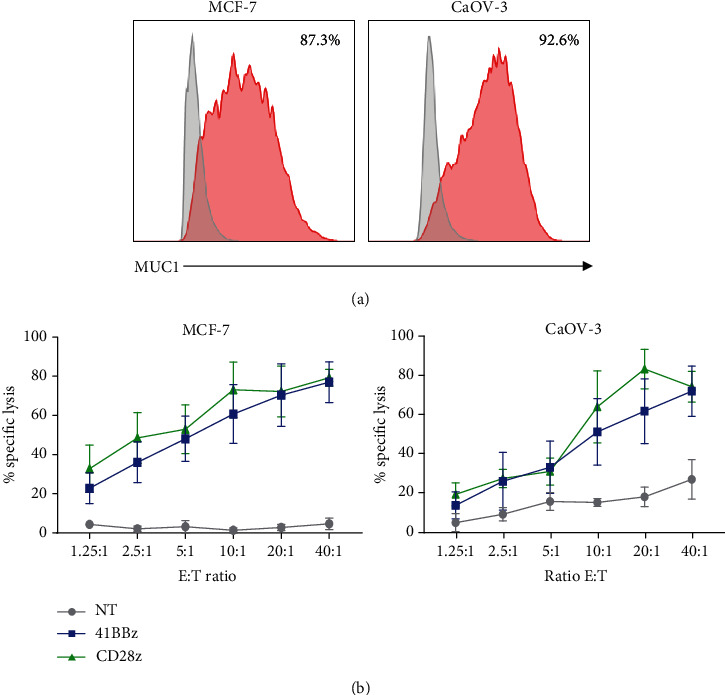
Specific lysis of CAR T cell targeting tumor associated MUC1. (a) Expression of MUC1 on different cancer cell lines, MCF-7 and CaOV3. (b) Six-hour cytotoxicity assay at E:T ratio 40 : 1, 20 : 1, 10 : 1, 5 : 1, 2.5 : 1, and 1.25 : 1. Target cells: MCF-7 (left) and CaOV3 (right). The data shown are mean ± S.E.M from four donors. Significance was determined by one-way ANOVA, ^∗^ for *P* < 0.05, ^∗∗^ for *P* < 0.01, and ^∗∗∗^ for *P* < 0.001.

In order to determine the antigen specificity and cytotoxic function of CAR MUC1 T cell, we performed a standard 6-hour cytotoxicity assay using two different cell lines, MCF-7 and CaOV3, which endogenously expressing MUC1 as targets. The cell surface expression of MUC1 was confirmed via flow cytometry (Figure 2(a)). The CAR.MUC1-41BB*ζ* and CAR.MUC1-CD28*ζ* T cells showed antitumor activity against two MUC1^+^ cancer cell lines in a dose-dependent manner, indicating a specific antigenic response of the two CAR constructs. CAR.MUC1-41BB*ζ* and CAR.MUC1-CD28*ζ* T cells had similar cytolytic activities against MUC1^+^ cancer cells which gradually increase from E:T ratio of 1.25 : 1 to 40 : 1. Both CAR.MUC1 T cells exhibited significantly higher cytolytic activities toward MUC1^+^ cancer cells compared with that of nontransduced T cells (Figure 2(b)). In addition, the cytolytic function of CAR MUC1 T cells was evaluated in primary breast cancer, in which both CAR MUC1 constructs demonstrated a specific antitumor activity and increased interferon gamma and IL-2 secretion after coculturing with MUC1^+^ primary cells (supplementary figure 1).

### 3.2. Immune Characteristics of CAR.MUC1-41BB*ζ* and CAR.MUC1-CD28*ζ*

**Figure 3:**
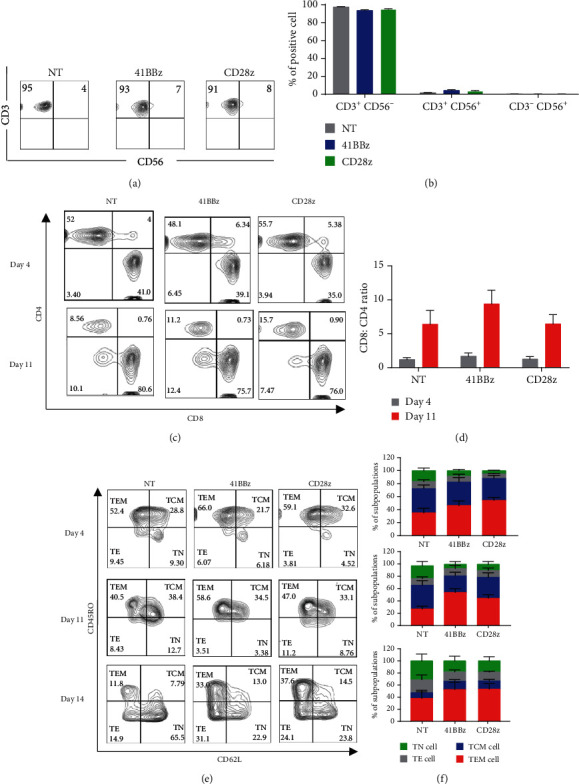
Comparison of immunophenotypes between CAR.MUC1-41BB*ζ* and CAR.MUC1-CD28*ζ*. The phenotypic analyses of CAR T cells were performed on days 4, 11, and 14 after transduction. (a) Representative flow cytometry plot demonstrating the cell subset analyzed with fluorochrome-conjugated anti-CD3 and anti-CD56 on day 4 after T cell transduction. (b) Bar graph represents the percentage of CD3^+^ T cells (mean ± S.E.M from 8 donors). (c) Representative flow cytometry plot at 4 and 11 after transduction. (d) Mean percentage of CD8^+^: CD4^+^ ratio on day 4 and day 11 after transduction is shown. Data shown are mean ± S.E.M from 6 donors. (e) Representative flow cytometry analysis of memory phenotype analyzed on days 4, 11, and 14 after transduction; CAR T cells were expanded in culture media containing IL-2 50 U/ml and stained with anti-CD45RO and anti-CD62L antibody (TEM: CD45RO^+^CD62L^−^, TCM: CD45RO^+^CD62L^+^, TN: CD45RO^−^CD62L^+^, and TE: CD45RO^−^CD62L^−^). (f) The mean percentage of naïve (TN), central memory (TCM), effector memory (TEM), and terminal effector (TE) T cells. Data represents as mean ± S.E.M (*n* = 6).

Next, we further characterize the immunophenotypes of CAR.MUC1-41BB*ζ* and CAR.MUC1-CD28*ζ* T cells. On day 4, all three groups contained more than 90% of CD3^+^ T cells (97.575% ± 0.265%, 93.625% ± 0.843%, and 94.9% ± 0.685% for nontransduced (NT), MUC1-41BB*ζ*, and MUC1-CD28*ζ*, respectively). Analysis of CD4 and CD8 subsets revealed that the ratio between CD8^+^ and CD4^+^ T cells was approximately 1 : 1 in all groups. However, after the expansion of T cells in culture with IL-2 for 1 week (day 11 after transduction), the percentage of CD8^+^ cytotoxic T cells increased in all groups (NT = 70.45% ± 9.63%, MUC1 − 41BB*ζ* = 74.27% ± 9.343%, and MUC1 − CD28*ζ* = 72.50% ± 7.773%), resulting in an increased ratio of CD8 to CD4 (Figures 3(a) and 3(b)).

As shown in Figure 3, on day 4 after transduction, all three groups exhibited a similar memory phenotype profile in which the majority of T cells were effector and central memory T cells, whereas the naïve T cell population was slightly higher in nontransduced T cells compared with both groups of CAR T cells (16.7% ± 3.9%, 8.5% ± 1.9%, and 5.01% ± 0.58% for NT, CAR.MUC1-41BB*ζ*, and CAR.MUC1-CD28*ζ* T cells, respectively). Analysis on day 11 following transduction showed that both CAR constructs contained approximately 30% central memory T cells (26.6% ± 5.95% for CAR.MUC1-41BB*ζ* and 33.6% ± 7.58% for CAR.MUC1-CD28*ζ*), a phenotype known to be correlated with *in vivo* persistence. However, after 14 days of culture, the T cells shifted toward effector (Figure 3(g)) and naïve (Figure 3(h)) T cells. We found a decrease in central memory T cells among the NT, CAR.MUC1-41BB*ζ*, and CAR.MUC1-CD28*ζ* population (Figure 3(f)). Therefore, we chose to harvest the cells on day 11 for subsequent experiments.

### 3.3. CAR.MUC1-41BB*ζ* and CAR.MUC1-CD28*ζ* Exhibit Potent Antitumor Activity In Vitro

**Figure 4:**
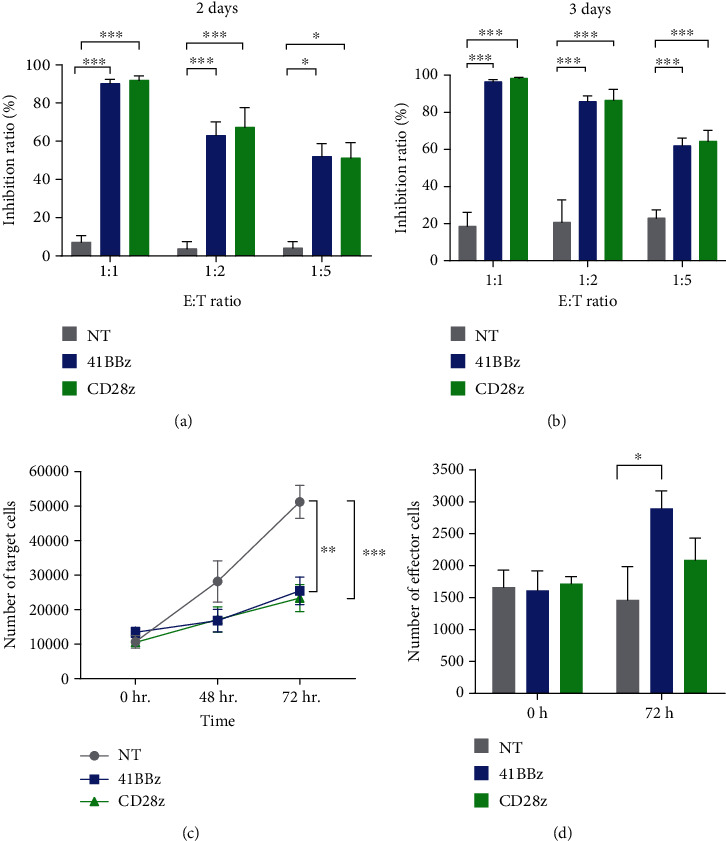
CAR.MUC1-41BB*ζ* and CAR.MUC1-CD28*ζ* exhibit potent antitumor activity in vitro. CAR.MUC1-41BB*ζ* and CAR.MUC1-CD28*ζ* were cocultured with MUC1^+^ breast cancer cell line, MCF-7 coexpressing eGFP at effector: target (E:T) ratio 1 : 1, 1 : 2, and 1 : 5 without adding cytokine for 2 and 3 days. At the indicated timepoint, a number of tumor cells and T cells were determined by flow cytometry using counting beads. The inhibition ratio of CAR.MUC1-41BB*ζ* and CAR.MUC1-CD28*ζ* after 2 days (a) and 3 days (b) coculture is shown. Data represents mean ± SEM (*n* = 6). Quantification of residual target cells (c) and effector cells (d) after coculture with CAR.MUC1-41BBz or CAR.MUC1-CD28z at ratio E:T of 1 : 5. Data represents mean ± S.E.M (*n* = 6). Statistical differences were analyzed by two-way ANOVA. *P* value significance is indicated as ^∗^ for *P* < 0.05, ^∗∗^ for *P* < 0.01, and ^∗∗∗^ for *P* < 0.001.

We next assessed the ability of CAR MUC1 T cells to expand and control breast cancer cells *in vitro*. MCF-7 eGFP^+^ cells were cocultured with CAR T cells at effector to target (E:T) ratios of 1 : 1, 1 : 2, and 1 : 5 for 2 and 3 days. The number of residual target and effector cells was analyzed via flow cytometry using counting beads, and the inhibition ratio was calculated as described in the methods. Both CAR.MUC1-41BB*ζ* and CAR.MUC1-CD28*ζ* exhibited potent antitumor activity after 2 days of coculturing with target cells (Figure 4(a)). After 3 days of coculture, we have observed a percent inhibition ratio of 85.9% ± 2.9% for CAR.MUC1-41BB*ζ* and 86.5% ± 5.8% for CAR.MUC1-CD28*ζ* at an E:T ratio of 1 : 2. The percent inhibition ratio was observed to decrease as the target ratio increased and was not different between the two CAR constructs (Figure 4(b)). Quantitation of residual tumor cells compared with pre-coculture numbers at an E:T ratio of 1 : 5 revealed that the number of MCF-7 cells cocultured with NT cells was significantly increased over time, whereas the second-generation CAR MUC1 containing either the 41BB or CD28 endodomain was able to control MCF-7 cell growth (Figure 4(c)). Interestingly, only CAR MUC1 T cells containing 41BB, but not CD28, exhibited T cell expansion after coculture with MUC1^+^ target cells (Figure 4(d)).

### 3.4. Incorporation of 41BB Exhibits Reduced PD-1 Upregulation after Antigen Stimulation

To further explore the differential effects of costimulatory 41BB and CD28 on antigen-induced T cell expansion, we examined CAR MUC1 T cells post coculture and found no significant difference in terms of CAR transgene expression between the two constructs. Assessment of the T cell phenotype also revealed that the memory phenotype of CAR.MUC1-41BB*ζ* and CAR.MUC1-CD28*ζ* after coculturing with MUC1^+^ tumor cells was not significantly differed. Additionally, we found that the activation marker, CD25, was significantly upregulated in both CAR.MUC1-41BB*ζ* and CAR.MUC1-CD28*ζ* but not NT cells, after antigen exposure, suggesting a similar activation state of CAR.MUC1-41BB*ζ* and CAR.MUC1-CD28*ζ* T cells (Supplementary figure 2).

**Figure 5:**
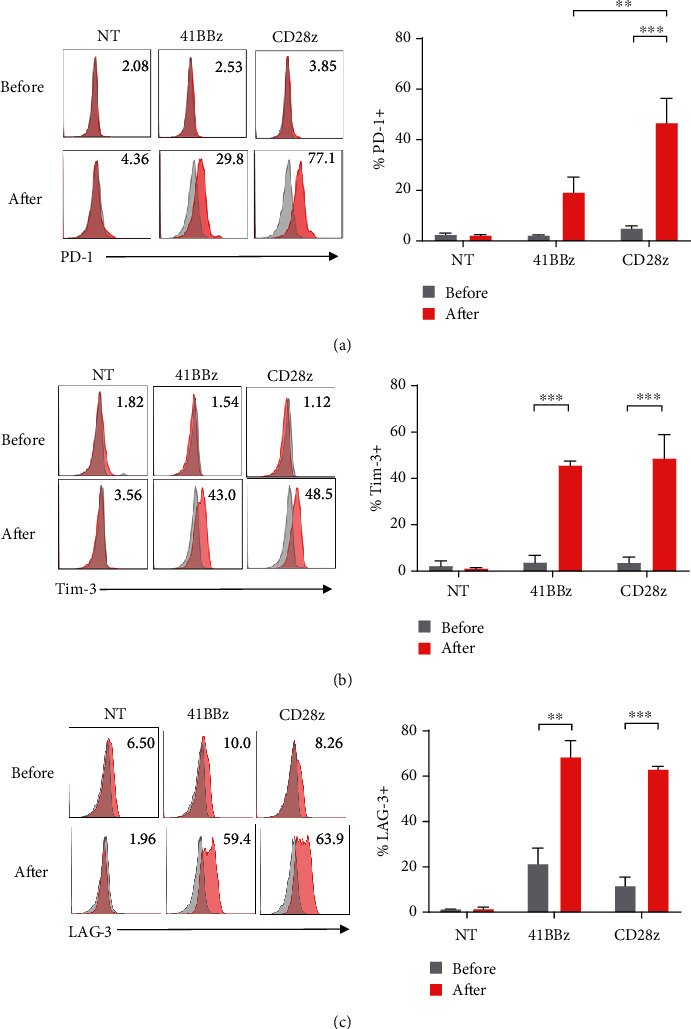
CAR.MUC1-41BB*ζ* exhibits less PD1 upregulation after antigen stimulation. (a–c) Representative histogram (left) and mean percentage (right) demonstrating the surface expression of PD-1 (a), TIM-3 (b), and LAG-3 (c) on nontransduced, CAR.MUC1-41BB*ζ* and CAR.MUC1-CD28*ζ* T cells before and after coculture with MUC1^+^ breast cancer cell line, and MCF-7 at ratio 1 : 1 for 72 h. Data represents as mean ± S.E.M (*n* = 6). Statistical significance was determined by two-way ANOVA and indicated as ^∗^ for *P* < 0.05, ^∗∗^ for *P* < 0.01, and ^∗∗∗^ for *P* < 0.001.

Thus, we have evaluated T cell exhaustion, which is identified as a key phenotype for T cell dysfunction and low persistence. The surface expression of PD-1, LAG-3, and TIM-3 on CAR T cells after coculturing with tumor cells was determined via flow cytometry. Both CAR MUC1 T cells significantly upregulated TIM3 and LAG3 expression after coculture with tumor cells (Figures 5(b) and 5(c)). Interestingly, PD1 expression was highly upregulated in CAR.MUC1-CD28*ζ* after antigen exposure, whereas a partial nonstatistically significant increase in PD1 expression was observed in CAR.MUC1-41BB*ζ* (Figure 5(a)), supporting our previous finding that antigen stimulated T cell expansion with the 41BB costimulatory domain, but not with CD28.

### 3.5. CD28 Is Associated with Upregulation of Suppressive Cytokines

**Figure 6:**
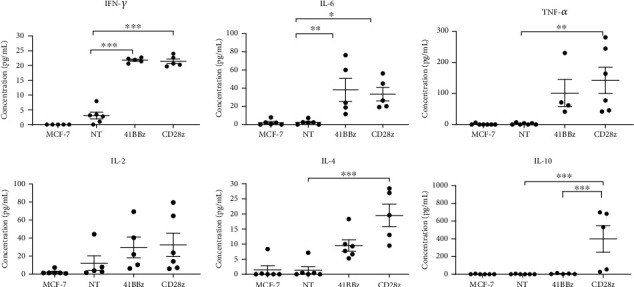
CD28 is associated with upregulation of suppressive cytokines. CAR.MUC1-41BB*ζ* and CAR.MUC1-CD28*ζ* were cocultured with MUC1^+^ breast cancer cell line at effector: target (E:T) ratio 1 : 1 without adding cytokine. Culture supernatants were collected and analyzed for cytokine concentration (IFN-*γ*, TNF-*α*, IL-10, IL-6, IL-4, and IL-2). Data represent as mean ± S.E.M (*n* = 6). Data were analyzed by one-way ANOVA. *P* value significance is indicated as ^∗^ for *P* < 0.05, ^∗∗^ for *P* < 0.01, and ^∗∗∗^ for *P* < 0.001.

Next, we investigated the cytokine profile of CAR MUC1 T cells after exposure to MUC1-expressing breast cancer cells. As shown in Figure 6, proinflammatory cytokine production, including IFN*γ* and IL-6, was significantly increased in CAR.MUC1-41BB*ζ* and CAR.MUC1-CD28*ζ* T cells compared with NT T cells. Further, we found that TNF-*α* expression was increased and statistically significant in CAR.MUC1-CD28*ζ* compared with NT cells (*P* = 0.0031). IL-2 was also increased in the culture supernatants of CAR.MUC1-41BB*ζ* (29.52 ± 11.56 pg/ml) and CAR.MUC1-41BB*ζ* (32.40 ± 12.92 pg/ml) compared with NT (11.93 ± 8.13 pg/ml), but it was not statistically significant. Interestingly, we observed a significant increase in the production of the immunosuppressive cytokine, IL-4, by CAR.MUC1-CD28*ζ*, but not CAR.MUC1-41BB*ζ* when compared with NT cells. Additionally, incorporation of the CD28 signaling domain has significantly increased IL-10 production compared with NT and 41BB*ζ* cells (*P* = 0.0029 vs. NT and *P* = 0.0047 vs. 41BB*ζ*).

## 4. Discussion

Chimeric antigen receptor T cells have been examined for their efficacy and safety in clinical trials for the treatment of breast cancer [9]. Here, we generated second-generation CAR T cell targeting tumor-associated mucin-1 (CAR MUC1) T cells and evaluated their antitumor activity *in vitro* against breast cancer. We found that two different costimulatory signals exerted a differential effect on the immunophenotype of CAR MUC1 T cells upon antigen stimulation. Incorporation of a costimulatory 41BB intracellular domain resulted in less upregulation of the exhaustion marker, PD-1, and decreased production of the suppressive cytokines, IL-4 and IL-10, when compared with CAR MUC1 T cell containing CD28 as a costimulatory signal.

Our CAR MUC1 T cells derived from anti-MUC1 antibody (HMFG2) targeting epitopes within the VNTR. This scFv has been used previously to target breast cancer and shown potent antitumor activity, while sparing its ability to discriminate between malignant and normal breast cells [21, 23]. Further study, however, has developed CAR T cells recognizing the altered glycosylated epitope within the MUC1 tandem repeat sequence (TAB004), which also demonstrated potent and selective antitumor activities in triple-negative breast cancer [19]. Evidence suggests that costimulatory domains have different effects on CAR T cell kinetics, immunophenotypes, and antitumor activities [24]. Among the various costimulatory molecules, we chose to investigate the antitumor activities and immunophenotypes of CD28 and 41BB as they have different target signaling pathway to initiate T cell activation. CD28 activation often results in increased T cell proliferation as well as IL-2 and Th1 cytokine production through PI3K/AKT pathway [20], whereas 41BB (CD137) promotes signal transduction via TRAF mediated NF-*κ*B, which then induces T cell proliferation and activation [25, 26].

Memory and effector cell differentiation influences T cell proliferative capacity, antigenic responses, and persistence. Evidence suggests a favorable anticancer response of CAR T cells with a less differentiated phenotype such as naïve (TN) or central memory T cells (TCM) [27]. Previous studies evaluating CD19-targeted CAR T cells in B cell malignancies demonstrated that the incorporation of 41BB into CAR constructs promoted the enrichment of the TCM population, whereas CAR with CD28 yielded more effector memory T cells (TEM), because of the different metabolic demands [28] and strong T cell signaling of CD28 [29]. However, in our study with CAR MUC1 T cells, we did not observe differences in T cell differentiation between the CD28 and 41BB endodomain. In fact, we observed that both CAR.MUC1-41BB*ζ* and CAR.MUC1-CD28*ζ* lost their naïve and central memory phenotype after ex vivo expansion in IL-2 for up to 14 days. This suggests that the degree of T cell differentiation is more associated with culture duration and conditions than the intrinsic costimulatory effects of MUC1 targeting CAR.

After antigen exposure, a similar antitumor activity of CAR MUC1 was noted, containing either 41BB or CD28. Moreover, the distinct costimulatory domain had no effect on the activation state of CAR MUC1 T cells as we observed a similar upregulation of CD25 and CAR transgene expression after coculturing with MUC1^+^ cancer cells. Indifference to antitumor activity *in vitro* was also reported with second-generation CAR targeting other antigens including B7H3 in pancreatic cancer [30] and PSMA in prostate cancer [31]. However, previous studies with CD19-targeted CAR demonstrated a greater T cell persistence and antileukemic activity with 41BB signaling [28, 32, 33]. We have also observed that our CAR MUC1 T cells containing a 41BB signaling domain exhibited better cell expansion after antigen stimulation and reduced PD-1 expression compared with CD28. Hui et al. demonstrated that upon ligation to PDL1, PD1 recruits the phosphatase, Shp2, which primarily dephosphorylates the CD28 costimulatory receptor and inhibits T cell activity [34]. Lower expression of PD1 in CD8^+^ T cells was also observed in mice treated with B7H3 CAR-41BB*ζ* T cells compared with CAR-CD28*ζ*[35]. Moreover, we have demonstrated that incorporating CD28 signaling in CAR MUC1 upregulated the immunosuppressive cytokines, IL-4 and IL-10, upon encountering the targeted tumor cells. This upregulation was neither observed nor prominent in CAR MUC1 containing 41BB. Previous evidence suggests suppressive activity of IL-4 on lytic activity and proliferation of cytotoxic T cells [36]. The presence of IL-4 in the tumor microenvironment was also found to be associated with tumor promotion and poor prognosis [37].

## 5. Conclusions

In conclusion, the type of costimulatory domain results in a different effect on the characteristics of CAR T cell targeting each antigen. The effect of costimulatory domains on CAR T cell characteristics is not universal and thus should be explored early for the development of the most suitable CAR construct for each target. We demonstrated that CAR MUC1 T cells containing the 4-1BB endodomain exhibited increased proliferation and reduced the upregulation of PD-1 with no upregulation of suppressive cytokine secretion after antigen stimulation compared with CAR MUC1 T cells containing a CD28 endosignaling domain. These characteristics could potentially improve the efficacy and persistence of anti-MUC-1 CAR T in breast cancer treatment. Further studies in breast cancer xenograft mouse models are necessary to compare *in vivo* antitumor activity and *in vivo* persistence between anti-MUC-1 CAR T cells with a 4-1BB or CD-28 endosignaling domain. Further investigations in the modification of CAR T cell to overcome suppressive elements in the tumor milieu should also be addressed to improve efficacy and safety of CAR MUC1 T cells for breast cancer treatment.

## Data Availability

The datasets used and/or analyzed during the current study are included in the article and available from the corresponding author on reasonable request.

## Supplementary Materials

Supplementary Materials Figure S1: specific lysis and cytokine production of MUC-1 CAR T cell with primary breast cancer cell. Figure S2: transgene expression, memory phenotype, and activation marker of CAR.MUC1-41BBz and CAR.MUC1-CD28z after antigen exposure.
