# Supplementary material for: An In Vitro Comparison of Costimulatory Domains in Chimeric Antigen Receptor T Cell for Breast Cancer Treatment
Source: J Immunol Res. 2022 Nov 22;2022:2449373. doi: 10.1155/2022/2449373 (PMC9708373; doi:10.1155/2022/2449373)
Supplement: Supplementary Materials — Figure S1: specific lysis and cytokine production of MUC-1 CAR T cell with primary breast cancer cell. Figure S2: transgene expression, memory phenotype, and activation marker of CAR.MUC1-41BBz and CAR.MUC1-CD28z after antigen exposure. [file 2449373.f1.docx]

**Supplementary Materials**

**Figure S1: Specific lysis and cytokine production of MUC-1 CAR T cell with primary breast cancer cell**

**
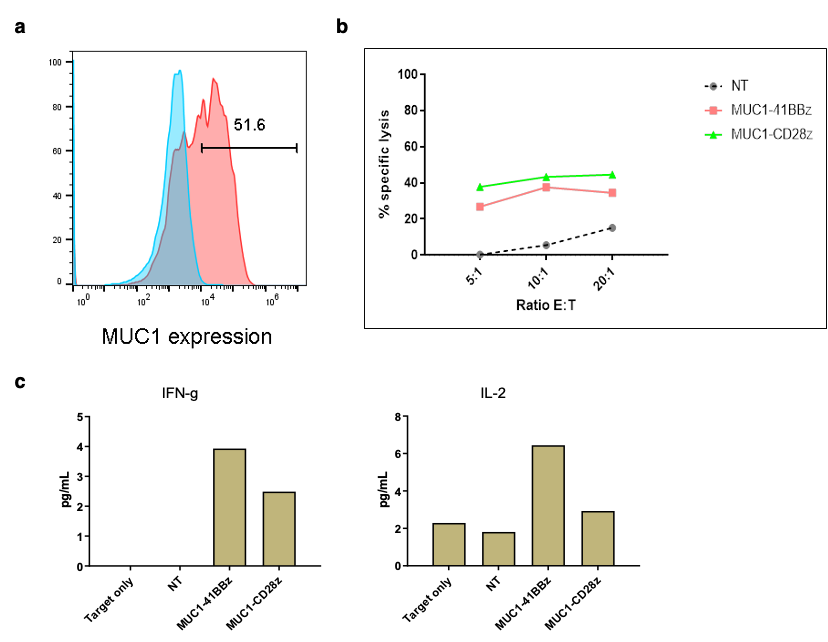

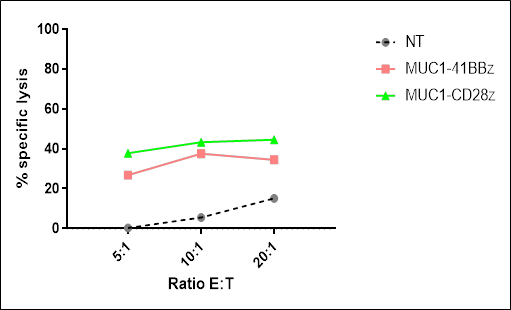
**

MUC1 expression

**a**

**b**

**c**

1. Expression of MUC-1 on primary breast cancer cell (n=1). Primary breast cancer cell exhibits high expression of MUC-1. (b) Six-hour cytotoxicity assay at E:T ratio 5:1, 10:1 and 20. Both CAR.MUC1-41BBz and CAR.MUC1-CD28z exhibit specific lysis toward primary breast cancer cell. (c) CAR.MUC1-41BBz and CAR.MUC1-CD28z were co-cultured with primary breast cancer cell at (E: T) ratio 1:1 without adding cytokine. Culture supernatants were collected and analyze for cytokine concentration (IFN-gamma and IL-2). Both CAR.MUC1-41BBz and CAR.MUC1-CD28z exhibit increase IFN-gamma and IL-2 production after coculture with primary breast cancer cell.

**Figure S2: Transgene expression, memory phenotype, and activation marker of CAR.MUC1-41BBz and CAR.MUC1-CD28z after antigen exposure**

**
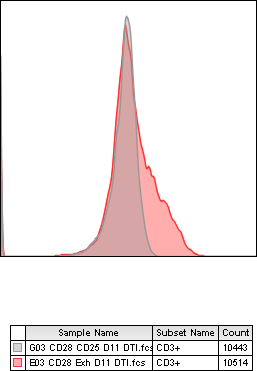

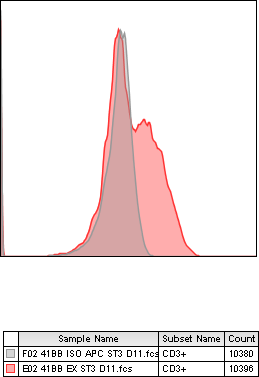

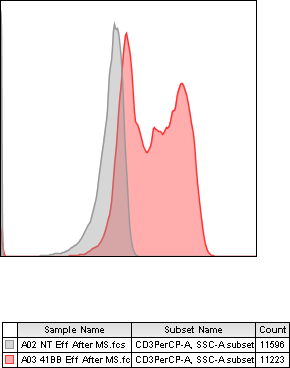

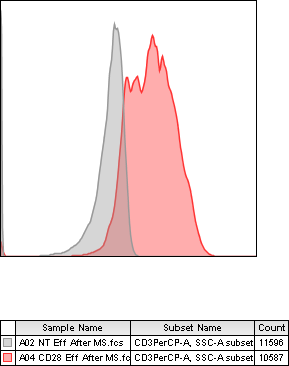

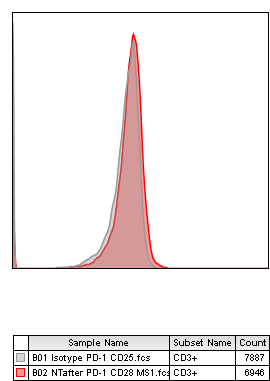

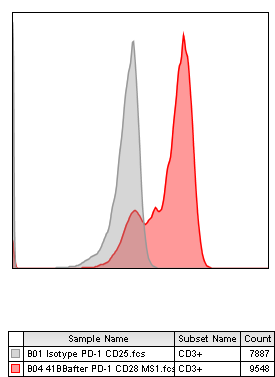

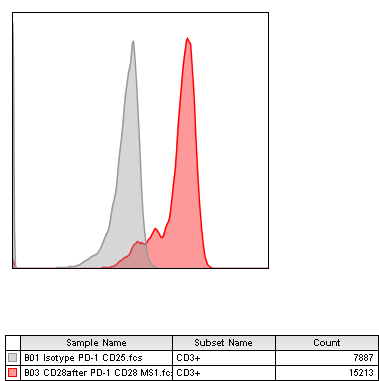

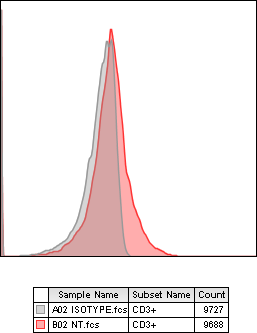
**

41BBz

CD28z

**a**

**b**

68.6

75.0

**NT**

**41BBz**

**CD28z**

**c**

Before

After

CAR

CD25

1.55

77.4

88.4

15.2

32.2

17.9

%CD25+

NT

41BBz

CD28z

**c**

CAR.MUC1-41BBz and CAR.MUC1-CD28z were co-cultured with MCF-7 cells at Effector: Target (E: T) ratio 1:1 without adding cytokine for 72 hour. **(a)** Representative flow cytometry demonstrating CAR expression following 72 h co-culture compared to pre-co-culture. **(b)** Mean percentage of transduction efficiency before and after co-culture Significance was determined by unpaired t test. Data are shown as mean ± S.E.M (n = 6). **(c)** and **(d)** Memory phenotype of CAR T cells following 72 h co-culture compared to before co-culture. Data represent as mean ± S.E.M (n = 6). **(e)** Representative histogram demonstrating surface expression of CD25 on CAR T cells following 72 h co-culture compared to before co-culture. (f) Mean percentage of surface expression of CD25. Data represent as mean ± S.E.M (n = 6). Significance was determined by two-way ANOVA, * for *P*<0.05, ** for *P*<0.01, *** for *P*<0.001.
